# Using Sex Pheromone and a Multi-Scale Approach to Predict the Distribution of a Rare Saproxylic Beetle

**DOI:** 10.1371/journal.pone.0066149

**Published:** 2013-06-19

**Authors:** Najihah Musa, Klas Andersson, Joseph Burman, Fredrik Andersson, Erik Hedenström, Nicklas Jansson, Heidi Paltto, Lars Westerberg, Inis Winde, Mattias C. Larsson, Karl-Olof Bergman, Per Milberg

**Affiliations:** 1 IFM Biology, Conservation Ecology Group, Linköping University, Linköping, Sweden; 2 Department of Plant Protection Biology, Swedish University of Agricultural Sciences, Alnarp, Sweden; 3 Department of Applied Science and Design, Mid Sweden University, Sundsvall, Sweden; Università degli Studi di Napoli Federico II, Italy

## Abstract

The European red click beetle, *Elater ferrugineus* L., is associated with wood mould in old hollow deciduous trees. As a result of severe habitat fragmentation caused by human disturbance, it is threatened throughout its distribution range. A new pheromone-based survey method, which is very efficient in detecting the species, was used in the present study to relate the occurrence of *E. ferrugineus* to the density of deciduous trees. The latter data were from a recently completed regional survey in SE Sweden recording >120,000 deciduous trees. The occurrence of *E. ferrugineus* increased with increasing amount of large hollow and large non-hollow trees in the surrounding landscape. *Quercus robur* (oak) was found to be the most important substrate for *E. ferrugineus*, whereas two groups of tree species (*Carpinus betulus, Fagus sylvatica, Ulmus glabra, vs. Acer platanoides, Aesculus hippocastanum, Fraxinus excelsior, Tilia cordata*) were less important but may be a complement to oak in sustaining populations of the beetle. The occurrence of *E. ferrugineus* was explained by the density of oaks at two different spatial scales, within the circle radii 327 m and 4658 m. In conclusion, priority should be given to oaks in conservation management of *E. ferrugineus*, and then to the deciduous trees in the genera listed above. Conservation planning at large spatial and temporal scales appears to be essential for long-term persistence of *E. ferrugineus*. We also show that occurrence models based on strategic sampling might result in pessimistic predictions. This study demonstrates how pheromone-based monitoring make insects excellent tools for sustained feedback to models for landscape conservation management.

## Introduction

Temperate ecosystems in Europe have been heavily altered by human disturbances to the extent that natural ecosystems are now absent or fragmented over large areas [1]. In the absence of systems of habitat patches large enough to meet the needs of species restricted to that habitat, the species may go regionally extinct [2]. Woodland pastures with old hollow deciduous trees are among the habitats most seriously affected by fragmentation in recent centuries [1]. Several insects associated with this type of habitat are therefore threatened. *Elater ferrugineus* L. (Coleoptera: Elateridae), the red click beetle, is one of the species associated with wood mould of old hollow deciduous trees, and is thus negatively affected by the massive loss of hollow deciduous trees caused by forestry and changing agricultural practices [3]. This beetle is classified as vulnerable in the Swedish Red List [4]. It has great potential as an indicator species for beetle biodiversity dependent on hollow deciduous trees (Andersson et al. unpublished data). Hence, in conservation management, it might function as an umbrella species, and knowledge about its habitat requirements might be generally applicable to other species. It is, for example, considered a main predator on another umbrella species, *Osmoderma eremita* [5], [6].

The common methods used to capture *Elater ferrugineus* in the field are direct sampling of wood mould, pitfall traps placed inside the hollow trees or suspending window traps placed outside the hollow trees [3], [7], [8]. However, these methods required huge trapping effort to properly identify the presence of a species at a site. Furthermore, it is unclear to what extent results achieved from strategic surveys of a species only in suitable patches can be transferred to a landscape scale [9], [10]. Several studies carried out on *E. ferrugineus* show that odour-based non-destructive sampling, by means of female-produced sex pheromone, is much more efficient than traditional approaches [3], [6], [8], [11]–[12]. Another recent study reported that pheromone-baited traps caught *E. ferrugineus* at 19 of 47 potential sites, while window/pitfall traps indicated its presence at only five of these sites (Andersson et al. unpublished data). Hence, pheromone-based surveys clearly improve systematic studies about distribution and habitat requirements of the red click beetle.

In the present study, we adopted this non-destructive pheromone-baited trapping method to evaluate, at multiple spatial scales, the relationship between the occurrence of *E. ferrugineus* and habitat quality and availability [13]–[15]. By using this approach, it is possible to draw conclusions about the spatial scales at which conservation efforts may be most efficient [16], [17]. So far, rare beetles such as *E. ferrugineus* have been surveyed only at potential hotspots in the landscape, thereby potentially biasing conclusions. In contrast, in this study beetle trapping was realised systematically in the landscape, irrespective of densities of host trees. We also explored the relative importance of potential host tree species, and the relative importance of hollows in the host trees, with the aim to provide explicit recommendations about spatial scales at which practical conservation efforts should be most efficient for this vulnerable saproxylic beetle. We used three parallel survey strategies when selecting sites, in order to validate our models by predicting occurrences from independent and partly non-overlapping data sets. Finally, we compared two sampling strategies (systematic vs. strategic) within the same landscape in order to evaluate how the strategic approach may bias data.

## Methods

### Study Species

*Elater ferrugineus* is a threatened click beetle with a body size of 17–24 mm [18]. This species has a life cycle of four to six years depending on the abundance of prey in the breeding substrate [3]. Its larvae live in the hollows as a predator on the larvae of other saproxylic beetles such as *Osmoderma eremita* [3]. The adults are usually active from late June to mid-August [12], live for 2–7 weeks and do not overwinter [6]. *Elater ferrugineus*’ main habitat is old hollow deciduous trees. In Sweden, oaks (*Quercus robur*) are considered the main habitat [4], [19] but the species can also be found in other tree species such as elm (*Ulmus glabra)*, lime (*Tilia cordata*), beech (*Fagus sylvatica*), alder (*Alnus glutinosa*), maple (*Acer platanoides*) and ash (*Fraxinus excelsior*) [18]–[20]. The distribution of *E. ferrugineus* ranges from Spain to the Caucasus, and from Italy in the south to Sweden in the north [21]–[23].

### Study Area

**Figure 1:**
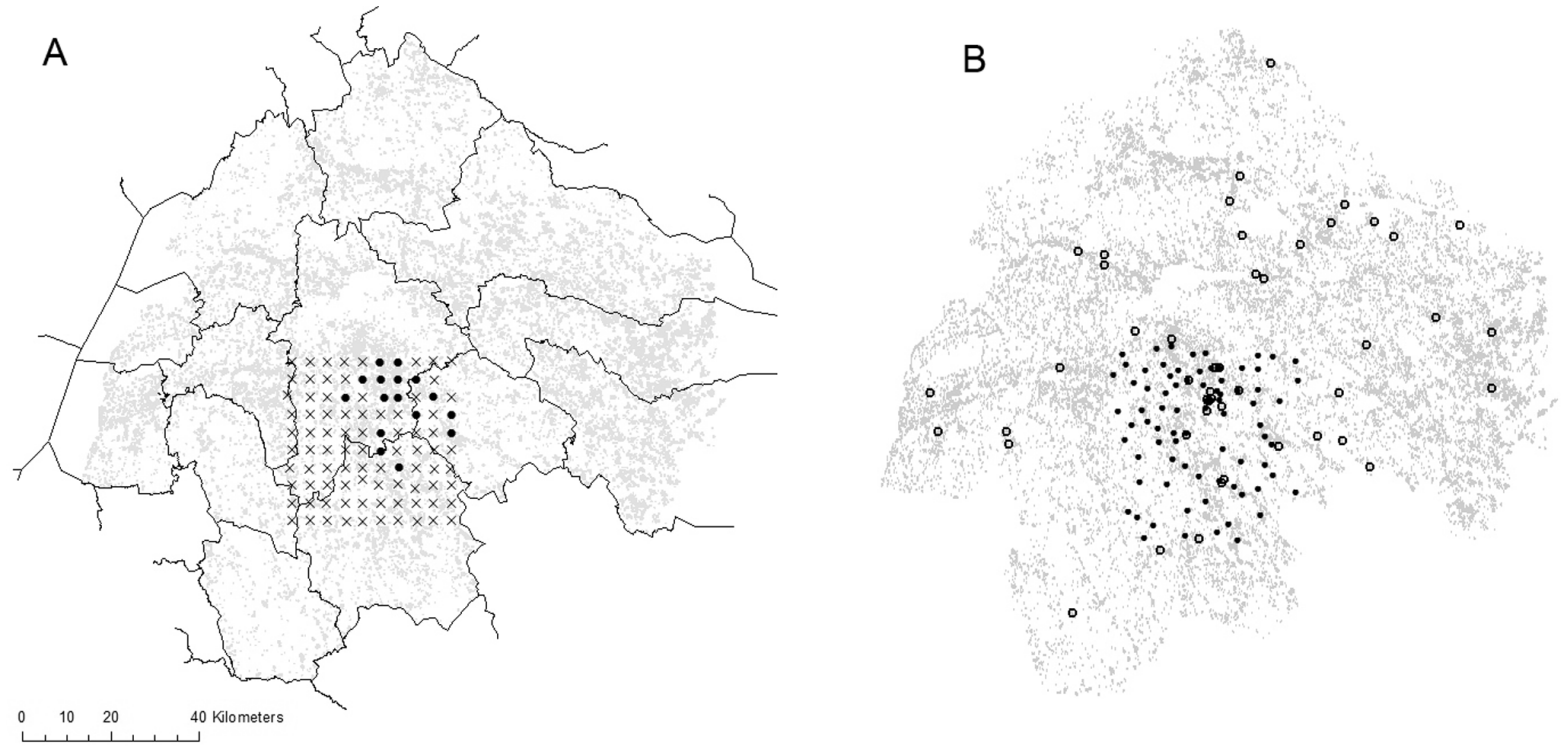
Trap locations in the county of Östergötland. (**a**) Trap location for systematically placed traps. Dots (•) represent occurrence of *E. ferrugineus* and crosses (×) represent non-occurrences. Thin lines delimit municipalities. (**b**) Trap locations of the two validation data sets. Filled circles (•) represent the strategic data set, sampled within the present study area and open circles (○) represent the Östergötland data set. Distributions of deciduous trees are marked in grey.

The study area was located in the county of Östergötland (Figure 1a), in the south-east of Sweden. The study area and its surroundings are dominated by arable land and coniferous forests. Deciduous forest stands with old/large and/or hollow trees were patchily distributed within the area.

### Beetle Sampling

The beetles were collected using a non-destructive odour-based trapping method, allowing specimens to be individually marked and released alive after every catch. Trapping was conducted from July 1^st^ to August 25^th^ 2011, which corresponds to the main flight period of the beetle. Permission for performing field work was obtained from landowners and permission to sample in protected sites also from the County Administration Board in Östergötland.

Two types of pheromone were used in this study to maximize detection probability of *E. ferrugineus.* Firstly, a compound resembling a pheromone produced by the female click beetle was used, which attracts males (7-methyloctyl (Z)-4-decenoate). Secondly, a pheromone emitted by males of their larval prey *O. eremita* ((*R*)-(+)-γ-decalactone) was used [3], [12]. This second compound attracts mainly female *E. ferrugineus* [6], [8], [11], [24]. 7-Methyloctyl (Z)-4-decenoate was synthesised according to a slightly modified procedure from [12] in >98.5% chemical purity and with a Z:E ratio of 90.7∶9.3 measured by GC and 92∶8 by NMR. Synthetic (*R*)-(+)-γ-decalactone was purchased from Sigma-Aldrich at an overall purity of 99% and an enantiomeric purity of 97%.

A 200 µL polypropylene polymerase chain reaction (PCR) tube containing 2 µL 7-methyloctyl (Z)-4-decenoate and a 2 mL glass vial containing 500 µL (*R*)-(+)-γ-decalactone were attached together using metal wire. An insect pin size 3 was used to pierce a hole in each PCR tube [3] and cotton dental rolls were inserted into the glass vials to allow compound release and to ensure a stable release rate throughout the whole trapping season [25]. Then, the PCR tube and glass vial were attached to a custom-built funnel trap [11]. The funnel trap was suspended by ropes from tree branches at 2–4 metres height on the most shaded side of the tree to prevent captured beetles from dying due to excessive heat. Trap were checked for the presence of the beetles every third day. Captured specimens in the trap were counted and given a unique marking on the elytra using a fine point permanent marker pen (Uni Paint Marker PX-21) before they were released. The beetles were marked in order to find out the number of unique individuals per trap. After an initial time period, traps without catches were visited more seldom (every sixth day). An occurrence in a trap was defined as a capture of at least one individual during the whole sampling period. The abundance per trap was defined as the number of unique individuals caught in each trap during the whole sampling period.

### Sampling Strategy

A systematic sampling strategy formed the core of this study. A total of 100 trap locations were placed in a 40 km × 40 km area (58° 0′ –22′N, 15°50′–16°4′E), with 4 km distance between traps (Figure 1a). The traps were placed in the nearest possible tree from the coordinates of the target locations; in a few cases, when the target location was in a lake or arable field, there was a substantial deviation. In a few cases, the placements were adjusted for logistic reasons. In one case, we failed to obtain access permission from the landowner.

In addition to systematic sampling, strategic sampling was used to obtain two validation data sets (Figure 1b): a) strategic sampling within the study area and b) strategic sampling in the entire county of Östergötland. These two data sets are hereafter referred as ‘strategically sampled data set’ and ‘Östergötland data set’, respectively. For the strategically sampled data set, 78 traps were placed mainly within the same 40 km × 40 km area, as for the dataset based on systematic sampling. Stands with large/hollow deciduous trees were selected in such a way that the stands represent a gradient from a single tree to sites with high numbers of large deciduous trees in the surroundings. The traps were placed in the centre of each site, and the minimum distance between traps was 500 m. For the Östergötland data set, 47 traps, that were part of a separate study (Andersson et al. unpublished data), were placed in ‘hot spots’ around Östergötland. These ‘hot spot’ sites were presumed to have the highest local diversity of oak-dependent saproxylic beetles in Östergötland (c.f. [15], [26], [27].

### Calculation of Tree Densities

We calculated the tree density in circular landscapes around each trap based on spatial data on large trees in the county of Östergötland, provided by the County Administration Board of Östergötland. The inventory for this data set was carried out between 1997 and 2008 [28]. The data set contains information on tree species, coordinates, circumference and hollow stages of each tree surveyed. The mature trees are also classified into one of five stages [28]: Trees without hollow are classified as stage 3, while trees with a hollow are classified as stage 4, 5, 6 or 7, depending on the size of the hollow (e.g. stage 4 is a tree with a cavity entrance approximately 5 cm diameter, and stage 7 is a tree with a large cavity reaching down to the ground). The total number of deciduous trees in the study area (49.4 km × 49.4 km) was 28,184 and these belonged to 19 taxa (Table 1). The study area consisted of the 40 km × 40 km grid with traps, and a buffer zone of 4.7 km including all trees within the radii of 4658 m (the maximum circle size used in this study). Nine per cent (2,482 trees) of the total number of trees were hollow trees ≥1 m diameter at breast height (dbh), 55% (15,572 trees) were hollow trees <1 m dbh, and 36% (10,130 trees) were non-hollow trees ≥0.70 m.

**Table 1:** Categorization of trees in the study.

Tree group	Hollow group	No of trees
***Quercus:***	>1 m dbh	1795
* – Quercus robur*	<1 m dbh	2965
	Non-hollow	4543
**Noble 1:**	>1 m dbh	93
* – Carpinus betulus*	<1 m dbh	370
* – Fagus sylvatica*	Non-hollow	1252
* – Ulmus glabra*		
**Noble 2:**	>1 m dbh	474
* – Acer platanoides*	<1 m dbh	3205
* – Aesculus hippocastanum*	Non-hollow	3261
* – Fraxinus excelsior*		
* – Tilia cordata*		
**Rosales:**	>1 m dbh	21
* – Malus* sp.	<1 m dbh	2210
* – Prunus avium*	Non-hollow	158
* – Pyrus communis*		
* – Sorbus aucuparia*		
* – Sorbus intermedia*		
**Malpighiales:**	>1 m dbh	85
* Populus* sp.	<1 m dbh	5453
* Populus tremula*	Non-hollow	604
* Salix caprea*		
* Salix* sp.		
**Fagales:**	>1 m dbh	14
* – Betula*	<1 m dbh	1369
* – Alnus glutinosa*	Non-hollow	312
Total		28184

Deciduous trees in the study area (49.4 km × 49.4 km) categorized according to the six tree groups and three tree hollow groups: ‘hollow’, which were then divided into two groups according to the diameter at breast height (dbh), and ‘non-hollow’ >0.70 m dbh.

In the present study, the trees with hollows were divided into two groups according to their diameter at breast height (dbh); hollow trees ≥1 m dbh and hollow trees <1 m dbh. All trees ≥0.7 m dbh without hollows were defined as non-hollow trees. Non-hollow trees with diameter <0.7 m dbh were not included in the study. Furthermore, all tree species were divided into six groups; *Quercus*, Noble 1, Noble 2, Rosales, Malpighiales and Fagales (excluding *Quercus*) (Table 1). The group *Quercus* contained only one species, i.e. *Q. robur*, as this tree species is considered the main preference for *E. ferrugineus* in Sweden [18] and is abundant in the study area. In Swedish legislation, there are 13 broadleaved tree species that are classified as ‘noble’ (*ädellöv* in Swedish) [29] of which seven were included in this study and of which several have been reported as a host to *E. ferrugineus* in Sweden [18], [19]. These seven were assigned into group Noble 1 or Noble 2 based on a combined consideration of plant phylogeny [30] and wood fresh weight (as a proxy for the durability of the timber to fungal and insect decay, using www.thewoodexplorer.com). Noble 1 was made up to species belonging to Eurosids I with higher wood fresh weight. Noble 2 was made up of four tree species that have lower wood fresh weight; three belong to Eurosids II and one to Euasterids I. The rest of the tree species were assigned into groups (Rosales, Malpighiales and Fagales) only according to phylogenetic placement.

### Data Analyses

We applied binomial generalized linear models (GLM; logit-link; software Statistica [31]) to relate the occurrence of *E. ferrugineus* at sites with tree density in the surrounding landscapes within circles of various sizes. The relative importance of these variables and the composition of tree groups were analysed to explain the occurrence of *E. ferrugineus*.

First, we tested for the effect of various tree hollow groups on the occurrence of *E. ferrugineus*, by including the density of trees within each of these groups and within each of the 31 different circle radii as an explanatory variable in the model. A tree hollow group was considered ‘important’ for *E. ferrugineus* if the occurrence of the beetle showed a significant positive relationship with tree density within at least one of the 31 different radii. Second, we tested the relative importance of the six tree groups: *Quercus*, Noble 1, Noble 2, Rosales, Malpighiales and Fagales. Furthermore, we calculated pair-wise Spearman Rank correlations between the densities of trees between the six different groups.

Based on the above analyses, densities for all ‘important’ tree groups were summed up and binomial GLMs were used to find out the characteristic scale of response [13], [15], i.e. the spatial scales (radius) where the relationship between occurrence and tree density was strongest (i.e. largest Wald value, provided the relationship was positive). The characteristic scales of response were used to calculate tree densities required for 25%, 50%, 75% and 90% probability of occurrence of *E. ferrugineus*. These tree densities, together with spatial tree data from the database provided by the county administration board, were used to predict the distribution of *E. ferrugineus,* using the Kernel density function in the software ArcGIS 10 [32].

To evaluate the models, we predicted the occurrence of *E. ferrugineus* in traps in the same area but strategically placed, and in traps spread throughout Östergötland, comparing predictions with occurrences in these two validation data sets. The sensitivity and specificity of the predictions, for different cutoffs, were then calculated and compiled in ROC (Receiver Operating Characteristics) curves.

Finally, to compare two trap placement strategies, model outcomes based on the 99 systematically placed traps were compared with those based on the 78 strategically placed traps within the same landscape. Both sets of models were built on the tree density of *Quercus* only, to facilitate comparisons with previous studies [15], [33]. Oak density needed for 50% probability of beetle occurrence was calculated for each radius and sampling strategy.

## Results

In this study, *E. ferrugineus* was captured a total of 46 times, including 10 recaptures, at 16 out of the 99 systematic traps (16%). In the first validation data set (strategically placed traps), *E. ferrugineus* was captured in 22 out of 78 traps (28%); in the second validation dataset (Östergötland data set), *E. ferrugineus* was captured in 19 out of 47 traps (40%).

### The Effect of Hollow Tree Stage for *Elater Ferrugineus*

**Figure 2:**
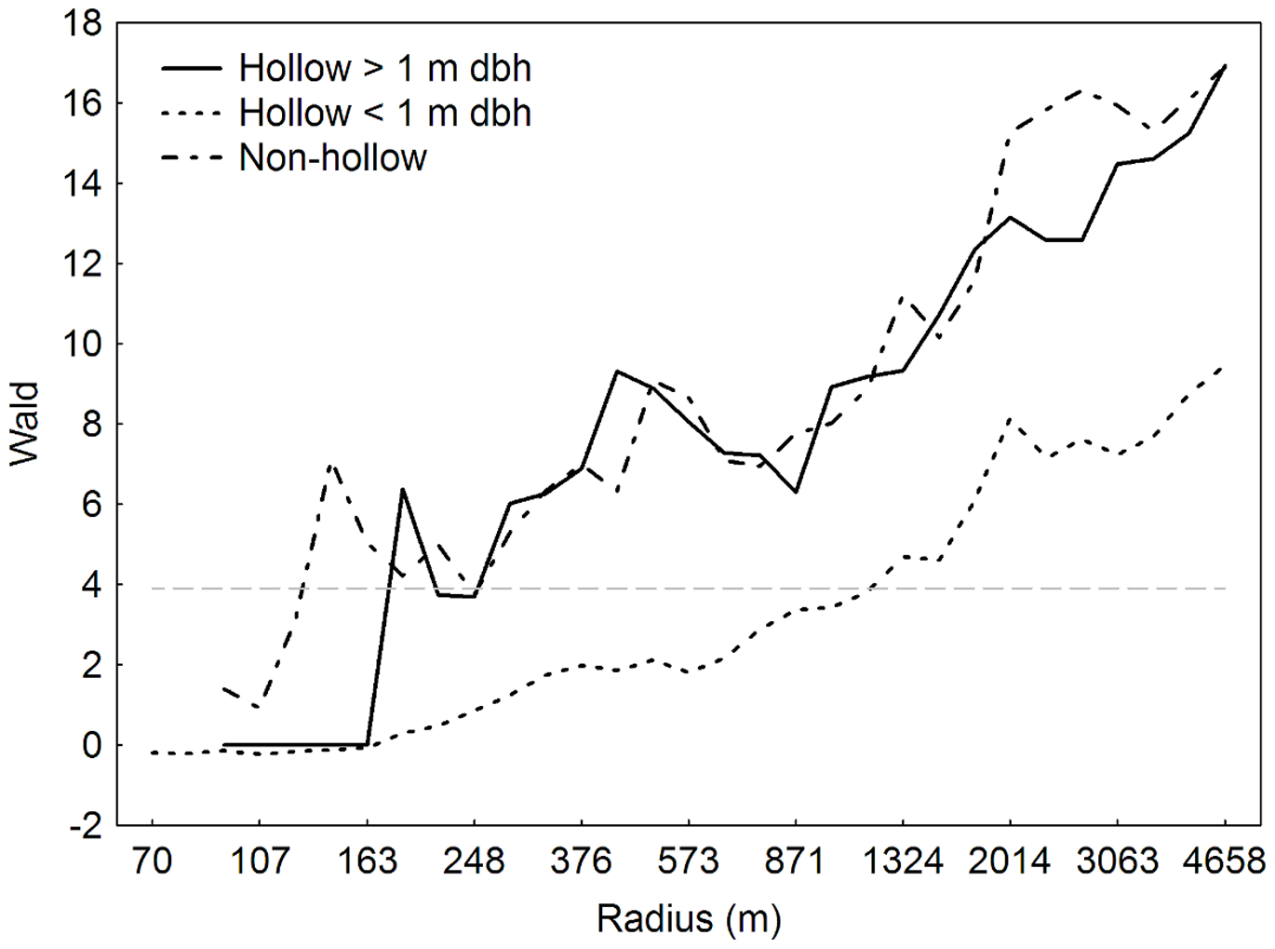
The relationships between occurrence of *Elater ferrugineus* and density of trees in different hollow classes. The relationships are expressed as Wald-values from 63 simple binomial GLMs. The explanatory variable ‘tree density’ is measured at 31 different spatial scales and includs trees from three different tree hollow classes: hollow trees ≥1 m dbh, hollow trees <1 m dbh and non-hollow trees. All models showed positive relationship between probability of occurrence and tree density. The grey line indicates p<0.05, corresponding to Wald value 3.9.

The occurrence of *E. ferrugineus* was significantly explained by the density of trees in all three tree hollow categories, and the relationships were increasingly stronger at larger spatial scales (Figure 2). The occurrence of *E. ferrugineus* was better explained by the density of large trees in the surrounding landscape, regardless of presence/absence of hollows in the trees, compared to small hollow trees. Since the density of trees within all three tree hollow groups significantly predicted the occurrence of *E. ferrugineus*, all tree hollow groups were included in calculating tree densities for the following analyses exploring the effect of tree groups (Table 1).

### The Effect of Tree Group for *Elater Ferrugineus*

The tree group *Quercus* constituted 33% (9303 trees) of the total number of trees included in the analyses, followed by Noble 2 with 25% (6940 trees) and Malpighiales with 22% (6142 trees). Noble 1 and Fagales were the smallest groups (1715 and 1695 trees, respectively).

**Figure 3:**
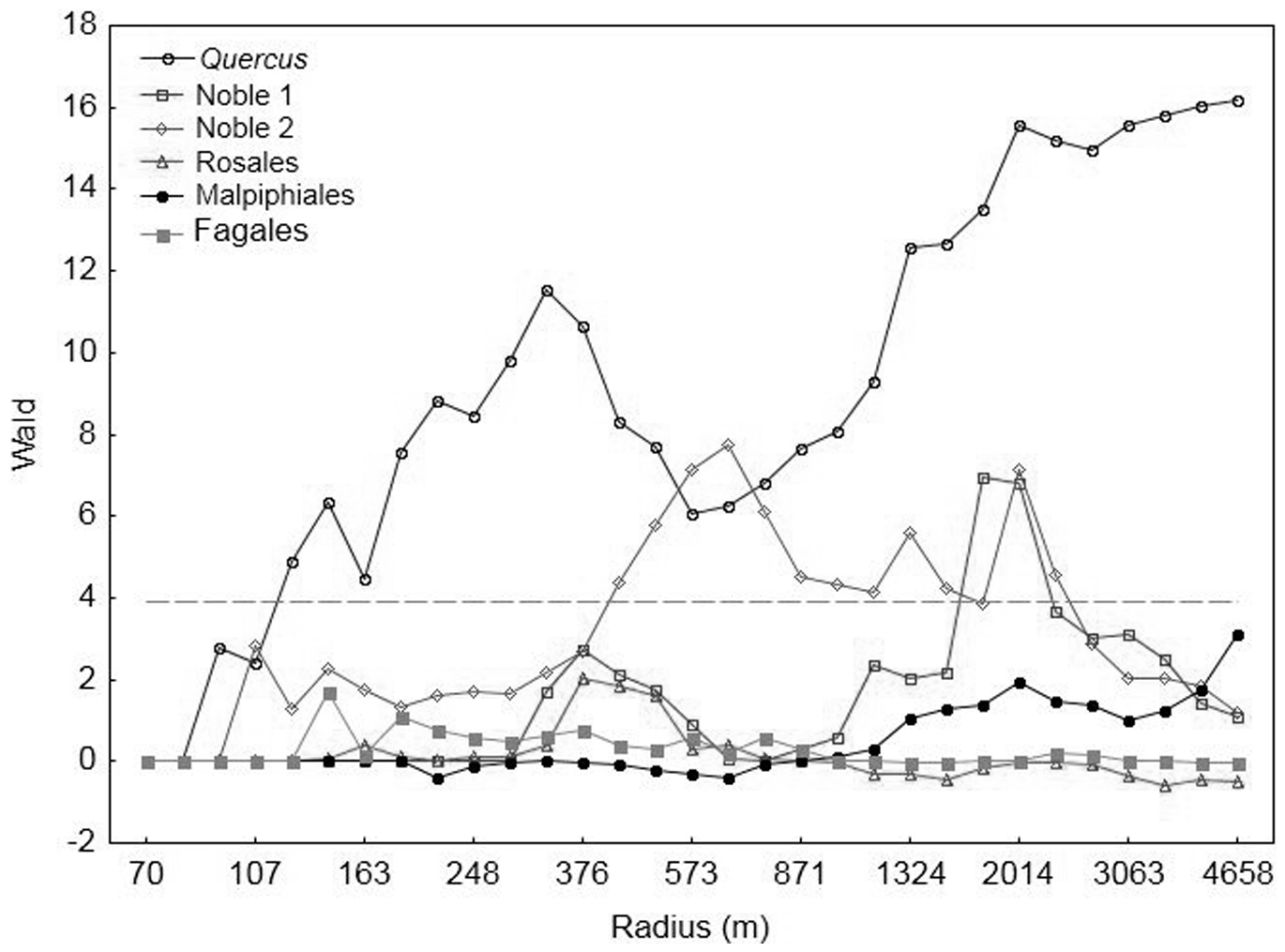
The relationships between occurrence of *Elater ferrugineus* and density of different species groups of trees. The relationships are expressed as Wald-values from 126 simple binomial GLMs. The explanatory variable tree density was measured at 31 different spatial scales and includs trees from six different groups. The grey line indicates p<0.05, corresponding to Wald value 3.9. The Wald statistic was given negative values in cases when it indicated a negative association between probability of occurrence and tree density.

The occurrence of *E. ferrugineus* increased with increasing density of trees in three out of six tree groups: *Quercus*, Noble 1 and Noble 2 (Figure 3). The occurrence was best explained by the density of *Quercus* followed by Noble 2 and Noble 1. The density of the three tree groups, Rosales, Malpighiales and Fagales could not explain the occurrence of *E. ferrugineus* (Figure 3).

### The Characteristic Scale of Response

**Figure 4:**
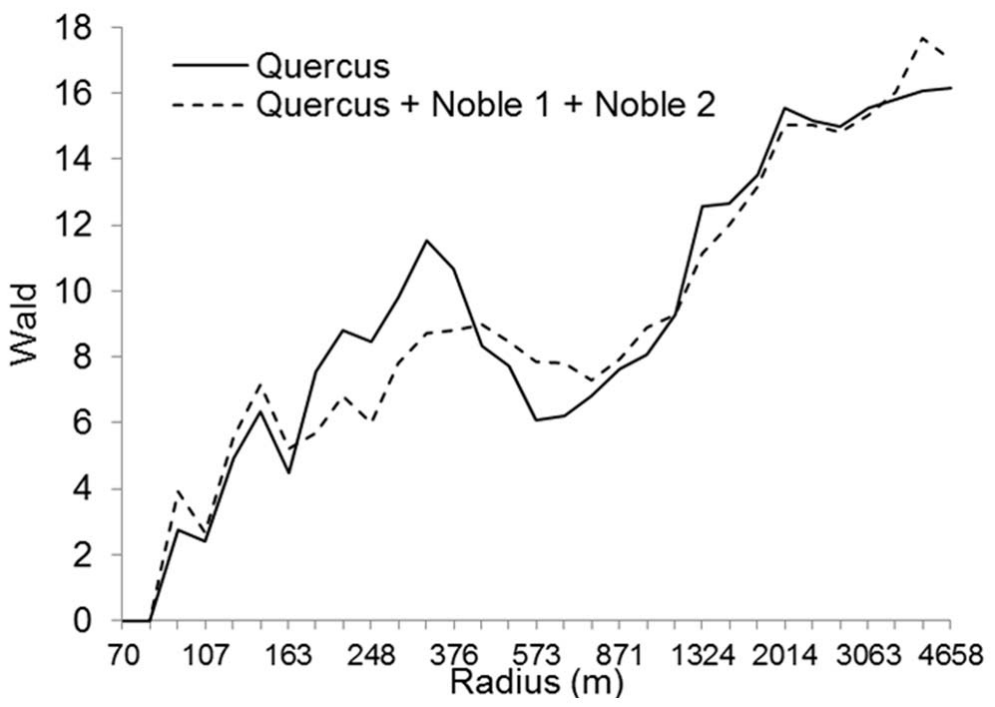
Characteristic scale of response for *Elater ferrugineus* using two models. i) Pooled density of trees within the groups *Quercus*, Noble 1 and Noble 2, and ii) density of *Quercus* only. The grey line indicates p<0.05, corresponding to Wald value 3.9.

Since the three tree groups *Quercus*, Noble 1 and Noble 2 significantly explained the occurrence of *E. ferrugineus*, the summed densities of these trees were used in further analyses. Figure 4 shows the relationships between the occurrence of *E. ferrugineus* and the pooled density of these three tree species groups at the 31 spatial scales. The occurrence of *E. ferrugineus* was best explained by the density of these trees within a circle radius of 4051 m. In addition, the occurrence was disproportionally well explained by the density of trees within a radius of 433 m, compared to the densities at other circle radii at about the same spatial scale. The Wald values in these analyses, however, were similar to the values in the analysis testing for the effect of *Quercus* alone (Figure 3), and in the analysis of *Quercus* alone, these two peaks were even more distinct. This means that *Quercus* alone, as well as in combination with Noble 1 and Noble 2, explained the occurrence of *E. ferrugineus* equally well, although there were minor differences in their impact at the intermediate spatial scales. In the model only including densities of trees in the group *Quercus*, the occurrence of *E. ferrugineus* was best explained at 327 m and 4658 m, the latter being the largest radius analysed (Figure 4). Since the two models predicted occurrences equally well, both models and their respective characteristic scales were used in model validations and predictions.

**Figure 5:**
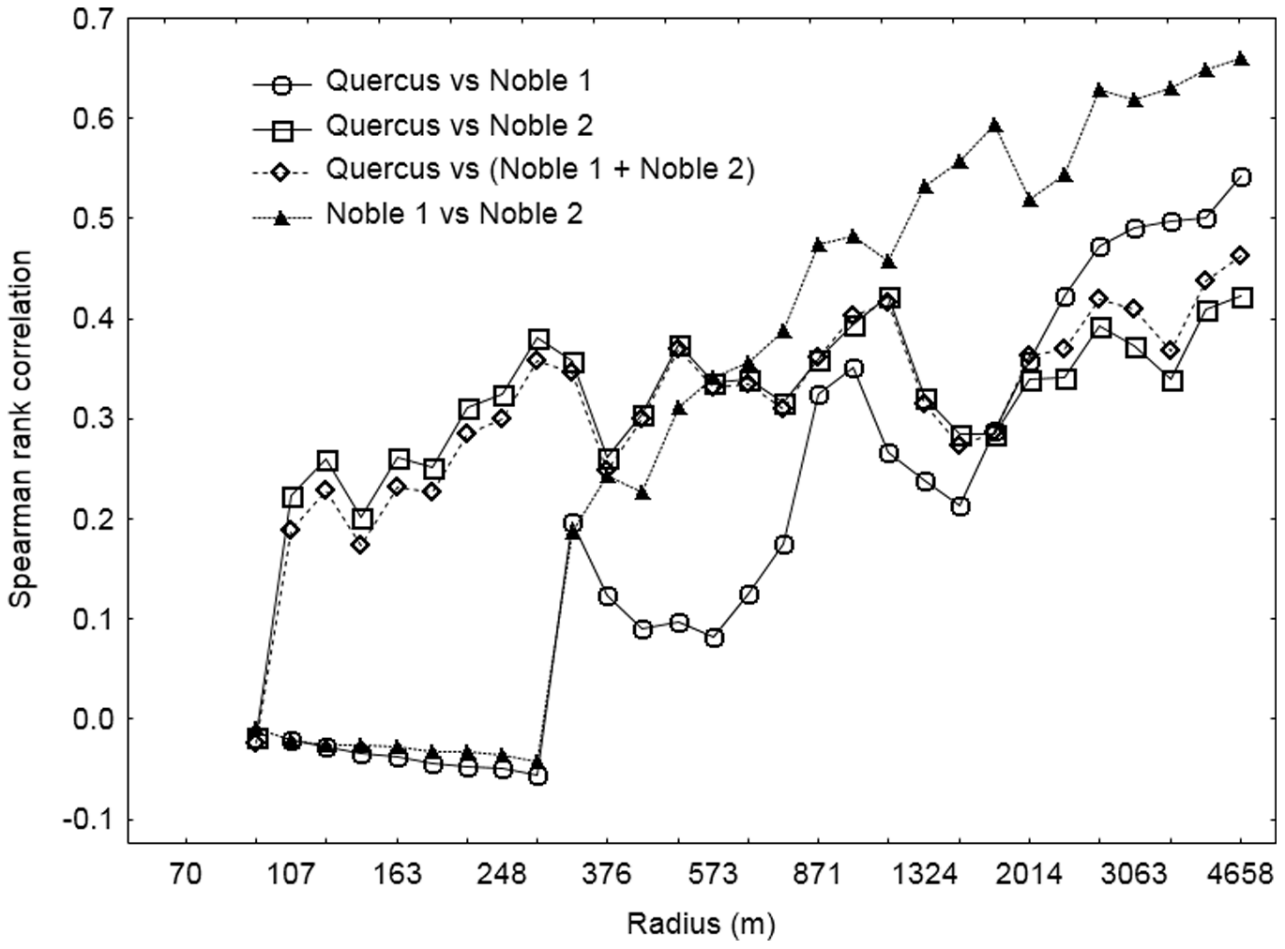
Correlation between the densities of tree groups that explained the occurrence of *Elater ferrugineus*.

The densities of trees in the groups *Quercus*, Noble 1 and Noble 2 were positively correlated to some extent (Figure 5).

### Prediction of Occurrences of Elater Ferrugineus

**Figure 6:**
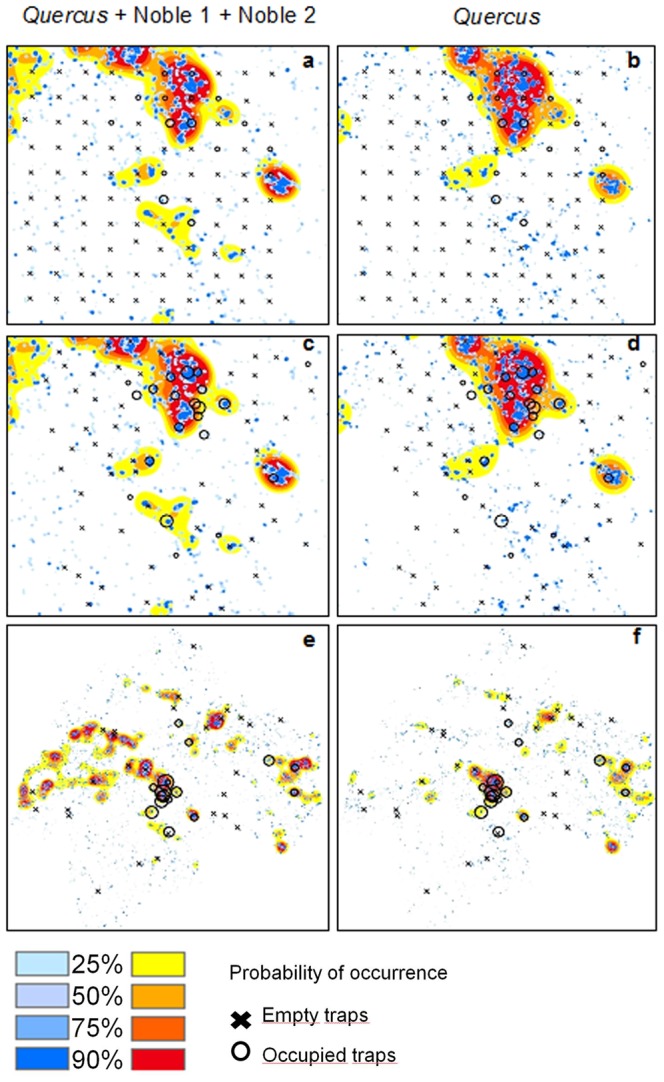
Maps of predicted occurrence of *Elater ferrugineus.* The maps show >25%, >50%, >75% and >90% probability of occurrence in the study area (**a,b,c,d**) and in the county of Östergötland (**e,f**) and the trap capture. Empty traps are presented by crosses (×) while occupied traps are marked with open circles (Ο) which size is proportional to the number of individuals caught. The first column (**a,c,e**) shows predictions based on models including the pooled density of trees within the groups *Quercus*, Noble 1 and Noble 2, while the second column (**b,d,f**) shows predictions based on models including the density of *Quercus* only. (**a,b**) shows trap captures in systematically placed traps, (**c,d**) the strategically sampled validation data and (**e,f**) the validation data set sampled in the entire Östergötland. In each map, the predictions are based on two models, one for each characteristic scales of response (blue tones represent a smaller scale: 433 m (pooled density of *Quercus*, Noble 1 and Noble 2) and 327 m (density of *Quercus* only), while orange tones represent prediction at larger scale: 4051 m (*Quercus*, Noble 1 and Noble 2) and 4658 m (*Quercus*).

Tree densities corresponding to 25%, 50%, 75% and 90% probability of occurrence of *E. ferrugineus* are shown in Table 2, for two alternative models and for two sets of characteristic scales of response (433 m and 4051 m for *Quercus*, Noble 1 and Noble 2 combined; 327 m and 4658 m for *Quercus* only). The predicted distributions of *E. ferrugineus* in the study area, based on the calculations in Table 2, are shown in Figure 6a and 6b.

**Table 2:** Tree density required for 25%, 50% 75% and 90% probability of occurrence of *Elater ferrugineus*, for two candidate models and for two characteristic scales of response.

Model	Characteristic scale of response (m)	Tree density (ha^–1^) requirement			
		25% occurrence	50% occurrence	75% occurrence	90% occurrence
*Quercus,* Noble 1, Noble 2	433	0.16	0.32	0.48	0.64
	4051	0.11	0.16	0.20	0.25
*Quercus*	327	0.10	0.19	0.28	0.37
	4658	0.07	0.11	0.15	0.18

**Figure 7:**
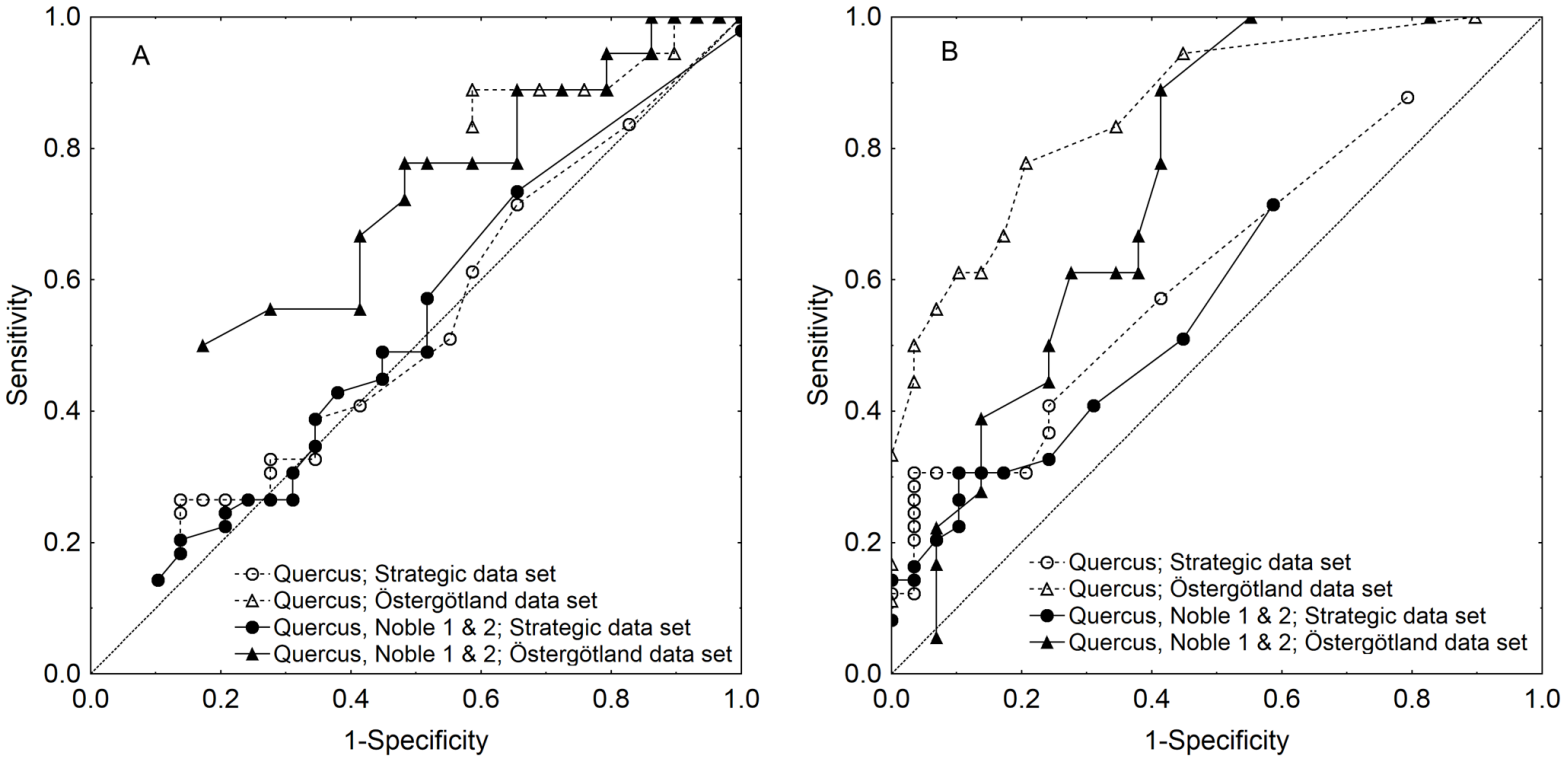
Sensitivity and specificity of two models in predicting the occurrence of *Elater ferrugineus* at trapping sites. The ‘strategically sampled data set’ consisted of data from the same study area (40 km × 40 km) as used in the main analyses, while the ‘Östergötland data set’ hotspot sampling over a larger geographic area. Models were either based on density of *Quercus* only, or by the combined density of *Quercus* and the tree groups Noble 1 and Noble 2.

When data of the beetle sampling from the present study were cross validated with the predicted occurrence for the two independent validation data set, most of the occupied traps were found to be located in the area that had the two characteristic scales of response overlapping (Figure 6c–f). The prediction maps also showed some sites where no beetle was caught but where the species had high probability of occurrence according to the model (Figure 6e, 6f). The ROC curves showed that large-scale models performed better, when applied to the validation data sets, than the small-scale models (Figure 7). In fact, small-scale models, especially when applied to the strategically sampled data set, did not seem useful (Figure 7a). Overall, the models better predicted the Östergötland data set (i.e. the hot spot sampling) than the strategically sampled one (Figure 7). The ROC curves suggested that sensitivity (ability to correctly classify occurrences) was more important for overall performance than specificity (ability to correctly classify lack of occurrence; Figure 7).

### Importance of Sampling Strategy

**Figure 8:**
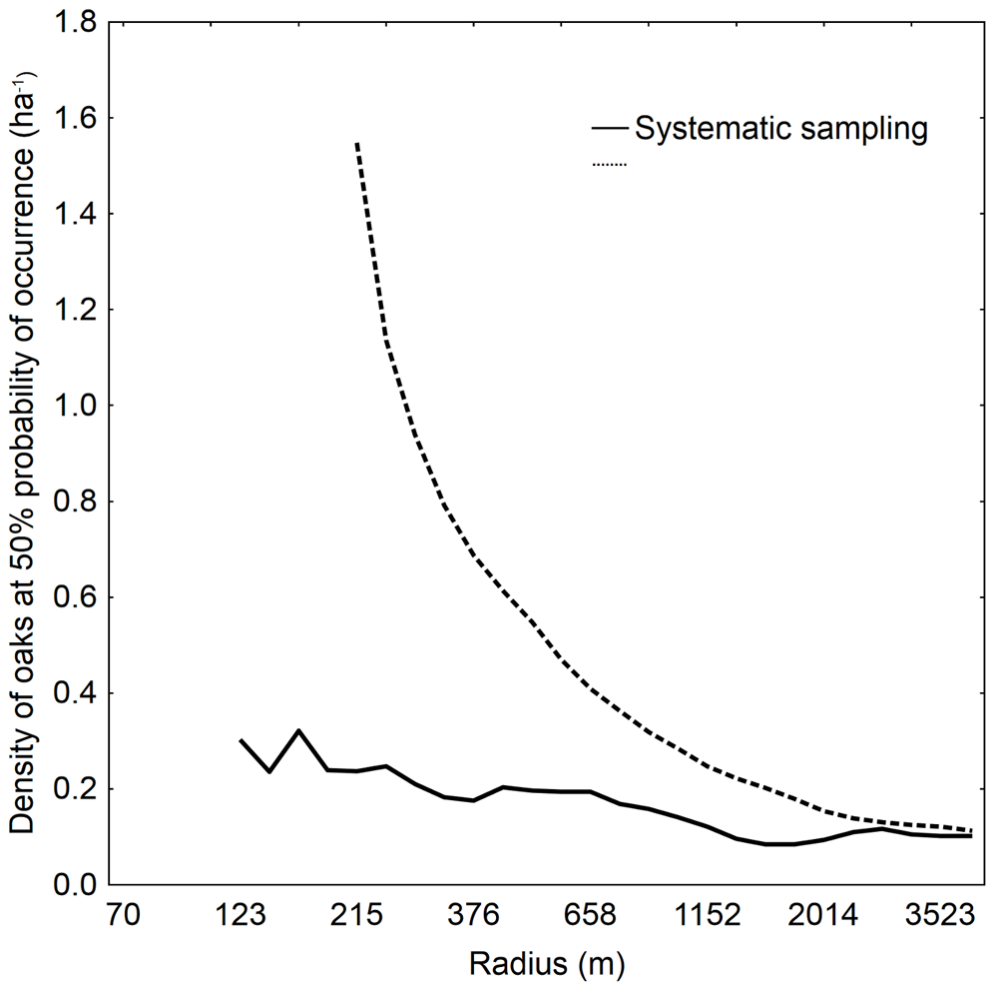
Model outcomes based on systematic sampling vs. strategic sampling. Smaller radii were excluded as such models were non-significant.

Comparing our two sampling strategies, it is apparent that predicted oak densities for 50% probability of occurrence differed substantially at smaller spatial scales, and that such differences decreased with increasing radius (Figure 8). Hence, when the larger characteristic scales identified above are considered, conclusions about oak densities needed for successful management of the beetle species would not differ. However, at the smaller characteristic scales identified, estimated density for 50% probability of occurrence were three times higher when based on strategic compared with systematic sampling (Figure 8).

## Discussion

Our study constitutes the first example of large-scale investigations of the landscape ecology of a rare and threatened insect with pheromone-based traps. The female-produced sex pheromone of *E. ferrugineus* apparently exhibits the legendary attractiveness commonly associated with insect sex pheromones [3], [12], allowing us to determine the true occurrence of this species with unprecedented accuracy. It seems very improbable that these pheromone traps might fail to detect local populations over a full flight season, with the possible exception of populations so small that they may not produce adults every year. An empty trap thus constitutes strong evidence for the absence of a local population, which for most insects can otherwise be notoriously difficult to demonstrate by means of stochastic trapping methods or directed surveys [7], [34]. This is especially pertinent for a species like *E. ferrugineus*, which has been considered a potential indicator for hollow-tree habitats with high conservation value, but for the caveat that it has been very difficult to survey and appears to be severely under-sampled with traditional methods [3], [7], [35], Andersson et al. unpublished data.

Pheromone-based trapping enabled us to survey *E. ferrugineus* populations at a great number of sites with minimal effort. This further permitted us to select sites according to three parallel strategies, in order to provide data sets for validation and to study whether the method for choosing sites could potentially skew the analysis. Such questions are relevant for many studies of rare insects, for which surveys and occupancy data could be expected to be preferentially skewed towards perceived hot-spots with high conservation value [36]. Our main set of traps, which was used to generate the predictive models, constituted an unbiased, systematic selection of trap sites with no prior relation to the distribution of hollow trees across the experimental area. The calculated densities for 50% probability of occurrence changed moderately depending on scale, dropping from 0.30 to 0.10 oaks ha^−1^ from the smallest to the largest radius considered (Figure 8). If, on the other hand, models were created from the “strategically sampled data set”, tree densities for 50% occurrence was substantially higher when considering small to intermediate scales compared with models based on the systematically samples data. An evident reason is that if the traps are placed in some of the best locations, this may inflate tree densities; whereas a systematic arrangement of traps may dispose them even in areas that lack trees. So, from a landscape perspective, our results show that a strategic hot-spot sampling (e.g. [15], [33]) will overestimate tree densities needed for saproxylic species and result in an overly pessimistic decision support for landscape planning. To what extent this conclusion is also relevant for other study systems remains to be established.

The strong significant relationship between the occurrence of *E. ferrugineus* and the density of *Quercus* indicates that *Quercus* is the main habitat of the beetle, and this is in accordance with what had been reported by [18], [19]. *Quercus* is probably a particularly important substrate for saproxylic beetles because the volume of wood mould is generally higher (on average older and larger trunks) and has a slower decomposition rate, compared to other tree species (e.g. [37]). Furthermore, it is the most species-rich tree genus with regard to saproxylic species [38]. The density of trees in the groups Noble 1 (*Carpinus, Fagus, Ulmus*) and Noble 2 (*Acer, Aesculus, Fraxinus, Tilia*) also explained the occurrence of the beetle, but the effect was weaker, especially for the tree species in the group Noble 1. This could partly be due to a lower proportion of trees in this group (6%), meaning lower statistical power in the analyses. Although the proportion of trees in the group Noble 2 was relatively high (25%, second after *Quercus* 33%), the proportionally stronger effect of the density of *Quercus* indicates that the occurrence of *E. ferrugineus* is highly dependent on available *Quercus*. As the density of the trees within the groups Noble 1 and Noble 2 significantly explained the occurrence of *E. ferrugineus*, and most of the tree species in these groups have been recorded as host for this species [18]–[20], these trees are probably a complementary host tree to *Quercus* for the beetle. In contrast, the occurrence of *E. ferrugineus* was not explained by the density of trees in the other three groups Rosales, Malpighiales and other Fagales. There are at least two possible reasons why they are not relevant: their age and size of trunks tend to be small, and their wood decomposition is often dominated by white-rot fungi (wood mould is mainly produced by brown rot fungi, together with other organisms). It should be noted that local cultural practices may strongly influence the availability of various species of hollow trees in the landscape, and thus determine which trees have the greatest impact at any given locality, as seen with the hermit beetle *O. eremita* in different parts of Europe [15], [39]–[41].

The occurrence of *E. ferrugineus* was better explained by the density of large hollow trees and large non-hollow trees than small hollow trees, i.e. the size of the tree seems to be more important for the beetle compared to presence of hollows. The larvae of *E. ferrugineus* develops exclusively in hollow deciduous trees [3], but the stronger effect of large hollow trees in contrast to the weaker effect of small hollow trees has not been documented earlier. In a study regarding saproxylic beetles, it has been shown that species richness in hollow trees increases as the tree girth increases, and hence with age [42]. Many beetle species may occur more frequently in old trees due to the higher quantity of wood mould in them [43] or due to the longer time span these trees have had for colonization events to occur. Wood mould begins to form in the trunks when the trees are about 150–250 years old [43], [44], which may have caused the relatively weak response by *E. ferrugineus* towards small hollow trees.

Unlike the strong relationship between *E. ferrugineus* and large hollow trees, the strong relationship between *E. ferrugineus* and large non-hollow trees was unexpected, even though the two types of trees tend to co-occur. All available information shows that *E. ferrugineus* is entirely dependent on pre-formed hollows as a larval substrate, which virtually excludes non-hollow trees as a habitat resource. One possible reason could be that large trees in general may contain cavities with entries too small to be noticed during the tree surveys. The number of tree individuals classified as [large] non-hollow in the data set was four times higher compared with the number of large hollow trees, and if these trees are used by *E. ferrugineus* to some extent, this could at least partly explain the effect. Perhaps more importantly, the current association between beetles and large trees in the landscape may match historical patterns of habitat resources, rather than their present-day distribution. The turnover of hollow oak habitats occurs over hundreds of years [42], which means that the current distributions of *E. ferrugineus* and different classes of trees in the landscape reflect events integrated over long time scales. Historical gaps in the recruitment of mature trees could have caused generational shifts leading to a recent time lag in the availability of hollow trees. To differentiate between these hypotheses would require comparisons between historical and present-day distributions of trees in the landscape.

The occurrence of *E. ferrugineus* increased with increasing densities of *Quercus* at both small and large spatial scales, with strongest relationships found with tree densities within the circle radii 327 m and 4658 m, respectively. A similar response to two spatial scales was found for the beetle *Tenebrio opacus* [15], where this dual response was interpreted as a response at two time scales. The smaller scale may reflect the static patches that *E. ferrugineus* needs to sustain its population at a short time scale. On a longer time scale, a large amount of substrate may be needed at a larger spatial scale to buffer against variations in the availability of hollow trees. It is important to note that the larger spatial scale identified in this study (4658 m) was in fact the maximum radius analysed, meaning that the true peak might be even larger.

When applying the models to the two validation data sets, predictions based on the smaller scales were disappointing while large scales worked well. This probably mainly reflects the differing sampling strategies that created data with higher tree densities as well as more occurrences in the validation data sets than in the data that generated the model. For the larger scales, the model including tree densities of *Quercus* worked better than the pooled tree density of the tree groups *Quercus*, Noble 1 and Noble 2, suggesting that an exclusively oak-based model would be preferred. The hotspot sampled Östergötland data set was well predicted (Figure 7), so transferability of models seem to be acceptable if the purpose is to identify potential hotspots in a landscape.

Surveys based on pheromone traps necessarily operate on the level of whole stands rather than individual trees. In light of the large characteristic scales of response identified in the present study, this seems like an entirely appropriate scale to understand the relationship between habitat resources and the landscape distributions of *E. ferrugineus*. Equivalent relationships, but based on occupancy of *E. ferrugineus* in individual trees [33], yielded a characteristic scale of response much lower than in the present study (1104 m vs. >4600 m; their models are too different from ours for a meaningful comparison of model predictions).

The predicted probabilities of occurrence could also be used to identify ostensibly suitable areas with high probability of occurrence, but which nevertheless currently lack *E. ferrugineus*. These areas appear suitable for attempts at re-introducing the species. However, rather than micro-managing single species it might be more useful to use it as a tool in the efforts to improve the overall landscape potential for re-colonization by saproxylic insects. If we consider the efforts applied to the current management of old oak stands relative to the increase of their timber volume (unpublished data), we should expect the start of a colonisation process. This might also be facilitated by establishing ‘corridors’ from current ‘hotpsots’ by using boxes with artificial wood mould [45]. Given the novel potential for pheromone-based monitoring of *E. ferrugineus* and other future model species, these would be very suitable as indicators over time to monitor the progress of re-colonization and provide feedback for models for sustainable landscape management.

To conclude, our results strongly demonstrate that a pheromone-based monitoring system constitute a potential game changer for conservation biology. This approach will/could greatly facilitate large-scale investigations of the landscape ecology of many rare and threatened insects with limited efforts. Pheromone traps for *E. ferrugineus* have allowed us to survey a large fraction of all potential habitat in the county of Östergötland in a single season. This has significantly extended our knowledge about the distribution of this elusive beetle, and yielded useful models to aid its long-term conservation. The conservation management of *E. ferrugineus* should give priority to areas with high density of old oaks, since these stands explain the occurrence of the beetle better than densities of other deciduous tree species (*Fagus, Ulmus, Acer, Aesculus, Fraxinus* and *Tilia*) which may act as complement to *Quercus*. The response of *E. ferrugineus* to the tree density at two separate scales indicates that a multi-scale approach might be essential in the conservation planning of this beetle.
